# Ohr Protects *Corynebacterium glutamicum* against Organic Hydroperoxide Induced Oxidative Stress

**DOI:** 10.1371/journal.pone.0131634

**Published:** 2015-06-29

**Authors:** Meiru Si, Jianbo Wang, Xiao Xiao, Jingyuan Guan, Yaoling Zhang, Wei Ding, Muhammad Tausif Chaudhry, Yao Wang, Xihui Shen

**Affiliations:** 1 State Key Laboratory of Crop Stress Biology for Arid Areas, Northwest A&F University, Yangling, Shaanxi, 712100, P. R. China; 2 Department of Biochemistry and Molecular Biology, College of Life Sciences, Northwest A&F University, Yangling, Shaanxi, 712100, P. R. China; 3 Environmental Analytical Laboratory, National Physical & Standards Laboratory, PCSIR, Islamabad, Pakistan; Instituto de Biociencias—Universidade de São Paulo, BRAZIL

## Abstract

Ohr, a bacterial protein encoded by the Organic Hydroperoxide Resistance (*ohr*) gene, plays a critical role in resistance to organic hydroperoxides. In the present study, we show that the Cys-based thiol-dependent Ohr of *Corynebacterium glutamicum* decomposes organic hydroperoxides more efficiently than hydrogen peroxide. Replacement of either of the two Cys residues of Ohr by a Ser residue resulted in drastic loss of activity. The electron donors supporting regeneration of the peroxidase activity of the oxidized Ohr of *C*. *glutamicum* were principally lipoylated proteins (LpdA and Lpd/SucB). A Δ*ohr* mutant exhibited significantly decreased resistance to organic hydroperoxides and marked accumulation of reactive oxygen species (ROS) *in vivo*; protein carbonylation was also enhanced notably. The resistance to hydrogen peroxide also decreased, but protein carbonylation did not rise to any great extent. Together, the results unequivocally show that Ohr is essential for mediation of organic hydroperoxide resistance by *C*. *glutamicum*.

## Introduction

Reactive oxygen species (ROS) are among the most potent threats to living organisms; ROS modulate the intrinsic balance between life and death [1]. ROS, including hydroxyl radicals, singlet oxygen, and hydrogen peroxide, are by-products generated via aerobic metabolic processes or upon stress caused by external agents [2]. When ROS levels are significant, the protective systems of living organisms are destroyed, and nucleic acids, proteins, carbohydrates, and lipids are damaged. In addition, pathogenic bacteria invading a host induce a burst of enzymatic ROS synthesis, and the host seeks to mount a defense [3]. Of the various types of ROS, organic hydroperoxides are particularly toxic, partly because they can generate free organic radicals, which in turn react with membranes and other macromolecules to promote free-radical chain reactions [4, 5]. Thus, protection against organic hydroperoxides is likely to play an important role in oxidative stress resistance.

Bacteria have evolved complex systems for sensing, protecting against, and regulating organic peroxide toxicity, to minimize the adverse effects of and repair damage caused by ROS [6]. Alkyl hydroperoxide reductase (Ahp), a member of the peroxiredoxin family, is one well-characterized enzyme involved in organic peroxide detoxification. The Organic Hydroperoxide Resistance (Ohr) protein exclusive to bacteria is another type of detoxification enzyme that specifically reduces organic peroxides to less toxic organic alcohols [7]. Ohr is a Cys-based thiol-dependent peroxidase that is structurally and functionally homologous to the osmotically inducible protein C (OsmC) [8]. Ohr contains two highly conserved redox-reactive cysteine residues located in motifs similar to those of the OsmC homolog; these residues play essential roles in peroxide metabolism [9]. During catalysis, one reactive cysteine is oxidized to a sulfenic acid intermediate, which is then immediately attacked by the second cysteine, triggering formation of a disulfide bond. Interestingly, lipoylated proteins (SucB, PDHB, and/or LpdA) serve as reducing systems that regenerate Ohr [10]. Upon *ohr* gene deletion, bacteria become specifically sensitive to organic peroxides, including cumene hydroperoxide (CHP) [11]. In fact, *ohr* deletion rendered the cells more sensitive to organic peroxides (specifically) than did deletion of AhpC in *Pseudomonas aeruginosa* [11]. Although Ohr has been well-studied in pathogenic bacteria including *Xylella fastidiosa* [12], *Mycobacterium smegmatis* [13], *Brucella abortus* [14], *Agrobacterium tumefaciens* [15], *Mycoplasma gallisepticum* [16], *Pseudomonas aeruginosa* [11], *Actinobacillus pleuropneumoniae* [17], and *Francisella tularensis* [18], little is known about the role played by the enzyme in bacteria of industrial importance.

*Corynebacterium glutamicum* is a widespread Gram-positive bacterium of industrial and environmental importance. The organism is metabolically versatile and grows on a variety of organic acids and/or sugars; the species is ecologically, medically, and economically important. *C*. *glutamicum* produces significant amounts of various L-amino acids, including L-lysine and L-glutamate, and vitamins [19]. However, during culture, *C*. *glutamicum* inevitably encounters unfavorable circumstances, such as low pH, high osmotic pressure, nutrient starvation, and/or oxidation [20]. Such challenges may compromise optimal growth and lower production [21]. One of the most serious problems is oxidative stress [22]. To counter this, *C*. *glutamicum* hosts several anti-oxidant systems. The bacterium produces large amounts of a low-molecular weight thiol, mycothiol (MSH; AcCys-GlcN-Ins), which is functionally equivalent to glutathione (GSH; γ-L-glutamyl-L-cysteinylglycine). Mycothiol is the principal non-enzymatic antioxidant countering external stressors including oxidative stress, alkylating agents, and antibiotics [21]. Also, *C*. *glutamicum* produces a battery of antioxidant enzymes including superoxide dismutase [23], catalase [24], thiol peroxidase [25], and mycoredoxin 1 (Mrx1) [26]; all act to prevent ROS-induced cell damage. Although the enzymes detoxifying superoxides and H_2_O_2_ have been well-studied [23, 24], much less is known about the defenses countering organic hydroperoxides. Therefore, we sought to explore the biochemical function of Ohr of *C*. *glutamicum* and to determine whether the enzyme plays a role in decomposing organic peroxides.

## Materials and Methods

### Bacterial strains and culture conditions

Bacterial strains and plasmids used in this study are listed in **S1 Table**. *C*. *glutamicum* and *Escherichia coli* strains were grown aerobically in Luria-Bertani (LB) broth on a rotary shaker (220 rpm) or on LB plates at 30°C and 37°C, respectively. Brain-heart infusion (BHI) broth supplemented with sorbitol (0.5 M) was used to generate mutants and maintenance of *C*. *glutamicum*. *C*. *glutamicum* RES167, a restriction-deficient strain derived from *C*. *glutamicum* ATCC 13032 was the wild-type background of all derivatives used in this study. Antibiotics were used at the following concentrations: kanamycin, 50 μg ml^-1^ for *E*. *coli* and 25 μg ml^-1^ for *C*. *glutamicum*; nalidixic acid, 40 μg ml^-1^ for *C*. *glutamicum*; chloroamphenicol, 20 μg ml^-1^ for *E*. *coli* and 10 μg ml^-1^ for *C*. *glutamicum*; ampicillin, 100 μg ml^-1^ for *E*. *coli*.

### DNA manipulation and plasmid construction

General DNA manipulations, transformations and agarose gel electrophoresis were carried out by applying standard molecular techniques. Primers used in this study are listed in **S2 Table**. PCR was performed with EasyTaq or EasyPfu DNA polymerase (TransGen Biotech, Beijing, China). Plasmids were isolated with plasmid DNA miniprep spin columns (TIANGEN, Beijing, China), and DNA fragments were purified from agarose gels by EasyPure Quick Gel Extraction Kit (TransGen Biotech, Beijing, China). Genes encoding *C*. *glutamicum* Ohr (NCgl0023, GI:23308767), dihydrolipoamide dehydrogenases (LpdA, NCgl0658, GI:19551918; Lpd, NCgl0355, GI:19551612), dihydrolipoamide acyltransferase (SucB, NCgl2126, GI:19553408), were amplified by PCR using *C*. *glutamicum* RES167 genomic DNA as template. These DNA fragments were digested by corresponding restriction enzymes and cloned into pET-28a and pET15b vectors to construct plasmid pET-28a-*ohr*, pET-28a-*lpdA*, pET-28a-*sucB* and pET15b-*lpd*, respectively. The plasmid pET-28a-*ohr* was used to generate the two Ohr site-directed mutants by overlap PCR [27] to replace active site Cys^60^ and Cys^124^ with Ser residue (Ohr:C60S and Ohr:C124S). Briefly, for C60S DNA construct, segments were amplified by PCR in two steps with mutagenic primers Ohr-F/Ohr-C60S-R and Ohr-C60S-F/Ohr-R (mutation sites are shown in bold in **S2 Table**) used to amplify segments 1 and 2, respectively. The second round of PCR was carried out by primer pair Ohr-F/Ohr-R using fragment 1 and 2 as templates to get *ohr*:*C60S* segment with desired mutation. The *ohr*:*C124S* DNA fragments were obtained by similar procedure with Ohr-F/Ohr-C124S-R and Ohr-C124S-F/Ohr-R primer pairs. *ohr*:*C60S* and *ohr*:*C124S* segments were digested and cloned into plasmid pET-28a to produce plasmids pET-28a-*ohr*::*C60S* and pET-28a-*ohr*::*C124S*, respectively. To construct the plasmid for *ohr* gene knock out, the 996-bp upstream PCR product and 980-bp downstream PCR product of *ohr* were amplified using primer pairs Dohr-F1/Dohr-R1 and Dohr-F2/Dohr-R2. Then, the upstream and downstream PCR fragments were fused together with primer pair Dohr-F1/Dohr*-*R2 by overlap PCR. The resulting DNA fragments were digested with BamHI/SalI and inserted into suicide vector pK18*mobsacB* to create pK18*mobsacB*-Δ*ohr*. For complementation of *ohr* in the Δ*ohr* mutant, *ohr* DNA fragments were digested and cloned into pXMJ19 vector to yield pXMJ19-*ohr*.

To construct the *lac*Z fusion reporter vector pK18*mobsacB*-*P*_*ohr*_::*lac*Z, overlap PCR was used to fuse the *ohr* promoter to the *lacZY* reporter gene [27]. Firstly, the 1,000 bp *ohr* promoter and the *lacZY* DNA fragment were amplified with the primer pair Pohr-F/Pohr-R and *lac*ZYF/*lac*ZY-R, respectively. Secondly, the two PCR products were used as the template with Pohr-F and *lac*ZY-R as primers, and the resulting PCR fragment was inserted into vector pK18*mobsacB* to get the pK18*mobsacB*-*P*_*ohr*_::*lacZ*. The fidelity of all constructs was confirmed by DNA sequencing (Sangon Biotech, Shanghai, China).

### Construction and complementation of an *ohr* deletion mutant strain in *C*. *glutamicum*

To construct the *C*. *glutamicum ohr* in-frame deletion mutant (Δ*ohr*), the plasmid pK18*mobsacB*-Δ*ohr* was transformed into *C*. *glutamicum* RES167 by electroporation, and chromosomal integration was selected by plating on LB agar plates supplemented with kanamycin. The Δ*ohr* deletion mutant was subsequently screened on LB agar plates containing 10% sucrose and confirmed by PCR and sequencing as previously described [28]. For complementation of *ohr* in the Δ*ohr* mutant, pXMJ19-*ohr* was transformed into the mutant strain by electroporation. The transformants were selected on LB agar plates supplemented with nalidixic acid and chloroamphenicol and *ohr* gene expression in *C*. *glutamicum* was induced by addition of 0.5 mM isopropyl-D-thiogalactopyranoside (IPTG) to the culture broth.

### Over-expression and purification of recombinant proteins

To obtain purified Ohr, SucB, Lpd, and LpdA proteins, *E*. *coli* BL21(DE3) transformed with pET-28a and pET-15b derivatives (**S1 Table**) were used for recombinant protein expression and purification. The recombinant strains were grown at 37°C in LB-kanamycin broth (*A*_600_ = 0.4–0.5), shifted to 22°C and induced by IPTG with final concentration of 0.5 mM. After grown for additional 12 h, the cells were harvested by centrifugation. The cell pellet was suspended in PBS and seven cycles of 30 s of sonication in ice were applied for cell disruption. The cell extract was centrifuged for 30 min to remove nucleic acid precipitates and final extract was purified with His·Bind Ni-NTA resin (Novagen, Madison, WI) according to manufacturer’s instructions. Recombinant thioredoxin (Trx), thioredoxin reductase (TrxR), Mrx1 and mycothione reductase (Mtr) proteins were prepared as reported previously [25]. Purified recombinant proteins were dialyzed against PBS at 4°C overnight and stored at -80°C until use.

### Peroxidase activity assay

The catalytic properties of Ohr with H_2_O_2_ and CHP as the substrate was determined as described previously [10] by monitoring the rate of NADPH oxidation. The reaction mixtures (300 μl) contained 50 mM sodium phosphate (pH 7.4), 50 mM NaCl, 1 mM DTPA (Diethylene triamine pentacetate acid, pH 7.4), 0.2 mM NADPH, 0.1 μM Ohr, an electron donor, and 1 mM peroxides(H_2_O_2_ or CHP). The Trx system (Trx 0–120 μM + TrxR 5 xM), Mrx1 (Mrx1 0–120 μM + Mtr 5 μM + MSH 500 μM), Lpd/SucB system (Lpd 0–120 μM + SucB 5 μM) and LpdA system (0–120 μM) were used as the electron donor system in the assays, respectively. All reactions were performed at 37°C and were initiated by the addition of H_2_O_2_ or CHP following 5 min pre-incubation, and oxidation of NADPH was monitored at 340 nm (*A*_340_). The amount of NADPH oxidized was calculated as μM s^-1^. Negative controls (without Mrx1, Trx, or peroxide) were run in parallel. The catalytic parameters for one substrate were obtained by varying its concentration at saturating concentrations of the other substrate (peroxide between 0 and 1 mM, or Trx, Mrx1, LpdA and Lpd 40 μM). The activity was determined after subtracting the spontaneous reduction rate observed in the absence of Ohr. Three independent experiments were performed at each substrate concentration.

Peroxidase activity was also analyzed by determining the consumption of peroxides with the ferrous iron xylenol orange (FOX) assay as previously described [29]. Reactions were initiated by the addition of thiol compounds and stopped at different intervals by addition of 20 μl HCl (1 M) into 100 μl reaction mixtures. The resulting mixture was mixed with 880 μl freshly prepared FOX reagent and incubated at 37°C for 20 min. The absorbance at 560 nm (*A*_560_) of each sample was determined after the color reaction had reached equilibrium.

### MALDI-TOF MS-MS analysis

Ohr was incubated with 10 mM DTT, 10 mM H_2_O_2_ and 5 mM CHP for 30 min at room temperature. The resulting proteins were subjected to nonreducing SDS-PAGE, and Coomassie brilliant blue stained bands were excised, in-gel digested with trypsin, and analyzed by MALDI-TOF MS-MS (Voyager-DE STR; Applied Biosystems).

### Ohr inactivation by NEM treatment

Recombinant His_6_-Ohr protein (2 mg/ml) was treated in 1 mM NEM for 1 h at room temperature and dialyzed against phosphate buffer (20 mM, pH 7.4) as described previously [12]. The concentration of His_6_-Ohr was determined by the Bradford Protein Assay Kit (Bio-Rad, Hercules, CA) with bovine serum albumin (BSA) as the standard.

### Determination of sulfenic acid formation

Determination of sulfenic acid (R-SOH) in Ohr proteins was performed by the TNB anion method described before [30]. TNB was known to react with sulfenic acids in a 1:1 stoichiometry, generating a mixed disulfide between TNB and a cysteine residue. Ohr wild-type (10 μM) and variants (10 μM) preincubated with H_2_O_2_ (50 μM) were treated with a 10-fold excess of TNB, prepared by incubation of an almost equimolar mixture of DTNB and DTT [12]. The amount of TNB remained (which was equal to the total TNB minus the consumed TNB) was determined spectrophotometrically.

### Sensitivity assays for oxidative agents

Efficacy of various environmental stress conditions was tested on *C*. *glutamicum* strains. Exponentially-grown *C*. *glutamicum* cultures (LB medium at 30°C) were diluted 100-fold with LB medium and various concentrations of oxidants were added into diluted cells before shaking at 30°C for 30 min. After treatment, the cultures were serially diluted and plated onto LB agar plates and colonies were counted after 36 h growth at 30°C. Percentage survival was calculated as follows: (CFU ml^-1^ of stressed cells/CFU ml^-1^ of cells without stress) ×100. All the assays were performed in triplicate.

### Measurement of intracellular ROS levels

2’,7’-dichlorofluorescein diacetate (DCFH-DA)-based assay described previously [31] was used to quantify levels of ROS *in vivo* with minor modifications. Briefly, aerobically grown cells (*A*_600_ = 1.6) were harvested by centrifugation, washed and resuspended in 50 mM PBS (pH 7.4) prior to pre-incubation with 2 μM DCFH-DA at 28°C for 20 min. Various concentration of stress inducers were added and incubated for another 30 min. Cells were washed twice with 50 mM PBS and resuspended in the same buffer. Fluorescence intensity was measured by Spectromax spectrofluorimeter (Molecular Devices, Sunnyvale, CA) with excitation at 502 nm and emission at 521 nm.

### Determination of cellular levels of protein carbonylation

Protein carbonylation assay was performed based on the method described previously [32] with minor modifications. Overnight-grown *C*. *glutamicum* strains were treated with various oxidants for 30 min with shaking at 30°C. Harvested cells were washed and resuspended in 25 mM Tris-HCl (pH 8.0) containing protease inhibitor cocktails (Sigma-Aldrich, St. Louis, MO), and sonication was performed to obtain a clear cell lysate. The soluble protein fraction was collected by centrifugation and protein concentration was measured by using the Bradford assay according to the manufacturer’s protocol (Bio-Rad, Hercules, CA). Protein carbonylation levels were detected with an OxyBlot Protein Oxidation Detection Kit (Millipore, Billerica, MA) based upon the manufacturer’s instructions, which measures carbonyl groups of proteins generated by oxidative reactions. Carbonyl groups in proteins were derivatized with 2,4-dinitrophenyl hydrazine (DNPH), and 20 μg of each DNPH-derivatized protein were loaded and electrophoresis was conducted on a 15% SDS-PAGE gel. After electrophoresis, DNPH-derivatized proteins were electroblotted onto nitrocellulose membranes, and immunodetection of DNPH-derivatized proteins was done using a rabbit antidinitrophenyl antibody (1:500; Millipore, Billerica, MA).

### MSH purification and determination

MSH was purified as previously described with thiopropyl sepharose 6B and Sephadex LH-20 chromatography and the concentration of purified MSH was determined with the thiol-specific fluorescent-labeling HPLC method using commercial GSH as the thiol standard [25].

### Construction of chromosomal fusion reporter strains and β-galactosidase assay

The *lacZ* fusion reporter plasmid pK18*mobsacB-P*_*ohr*_::*lacZ* was transformed into the wild-type *C*. *glutamicum* and the Δ*sigH* mutant by electroporation, and the chromosomal pK18*mobsacB-P*_*ohr*_::*lacZ* fusion reporter strains were selected by plating on LB agar plates supplemented with kanamycin [25]. β-galactosidase activities were assayed with ONPG as the substrate [33]. The β-galactosidase data represent the means of one representative assay performed in triplicate, and the error bars represent the standard deviation. Statistical analysis was carried out with Student’s *t*-test.

## Results

### *C*. *glutamicum* Ohr is a Cys-based thiol-dependent peroxidase

**Fig 1:**
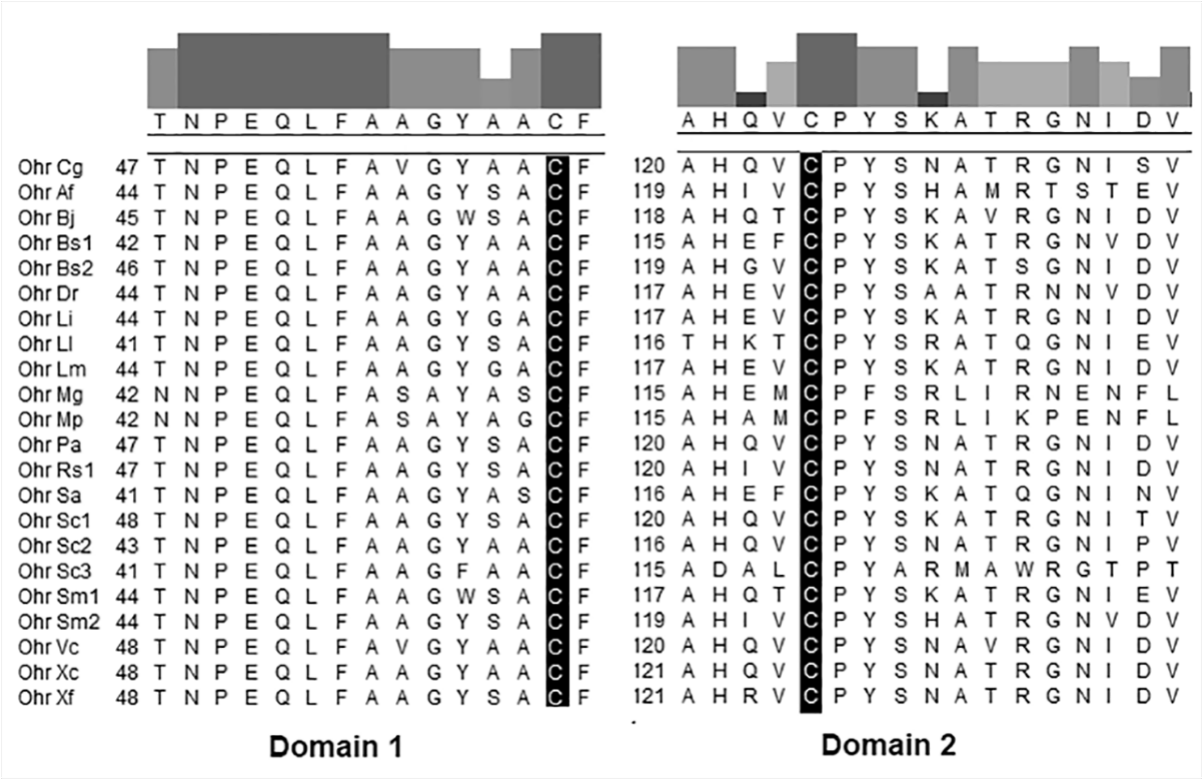
Alignment of conserved cysteine-containing domains of Ohr. The consensus was constructed using Clustal W of the MegAlign 5.00 software package (DNAstar Inc.); the Ohr proteins are derived from *Agrobacterium fabrum* C58 (Ohr Af, GI:15888188); *Bradyrhizobium japonicum* (Ohr Bj, GI:8708902); *Bacillus subtilis* 168 (Ohr Bs1, GI:16078381; Ohr Bs2, GI:16078379); *C*. *glutamicum* (Ohr Cg, GI:23308767); *Deinococcus radiodurans* (Ohr Dr, GI:499190952); *Lactococcus lactis ssp*. *lactis* (Ohr Ll, GI:15672574); *Listeria innocua* Clip11262 (Ohr Li, GI:16801366); *Listeria monocytogenes* EGD-e (Ohr Lm, GI:16804238); *Mycoplasma genitalium* (Ohr Mg, GI:148840409); *Mycoplasma pneumonia*e (Ohr Mp, GI:13508407); *Pseudomonas aeruginos*a (Ohr Pa, GI:15598046); *Ralstonia solanacearum* (Ohr Rs1, GI:17549328); *Staphylococcus aureus* ssp. *aureus* Mu50 (Ohr Sa, GI:15923818); *Streptomyces coelicolor* A3(2) (Ohr Sc1, GI:6562797, Ohr Sc2, GI:7546676, Ohr Sc3, GI:9885209); *Sinorhizobium meliloti* (Ohr Sm1, GI:16263744, Ohr Sm2, GI:15964715); *Vibrio cholerae* N16961 (Ohr Vc, GI:15601759); *Xanthomonas campestris pv*. *phaseoli* (Ohr Xc, GI:59799888); and *Xylella fastidiosa* (Ohr Xf, GI:15838425). The histogram shows the conservation strengths of residues in two relevant domains.

We identified the gene encoding a putative Ohr (NCgl0023, GI:23308767) by running a BLAST search and analyzing the genomic sequence. *Ohr* is located between base pairs 24,295 and 24,732 of the *C*. *glutamicum* genome (and is thus 438 nucleotides in length), and it encodes a protein of 145 amino acid residues with a theoretical molecular mass of 14.9 kDa. Ohr shares 53%, 46%, and 48% amino acid sequence identity with the Ohr proteins of *Vibrio cholerae*, *X*. *fastidiosa*, and *Deinococcus radiodurans*, respectively, and the *ohr* gene is present as a single copy (unlike the *ohr* genes of *Bacillus subtilis* [34] and *Streptomyces coelicolor* [35], both of which have 2–3 copies). Sequence homology analysis showed that *C*. *glutamicum* Ohr has two conserved Cys residues at positions 60 and 124, both of which are in domains highly conserved among Ohr homologs [12]. Cys^60^, bracketed by several hydrophobic residues, lies in hydrophobic domain 1, and Cys^124^ is located in a VCP motif of hydrophilic domain 2 [12] (**Fig 1**).

**Fig 2:**
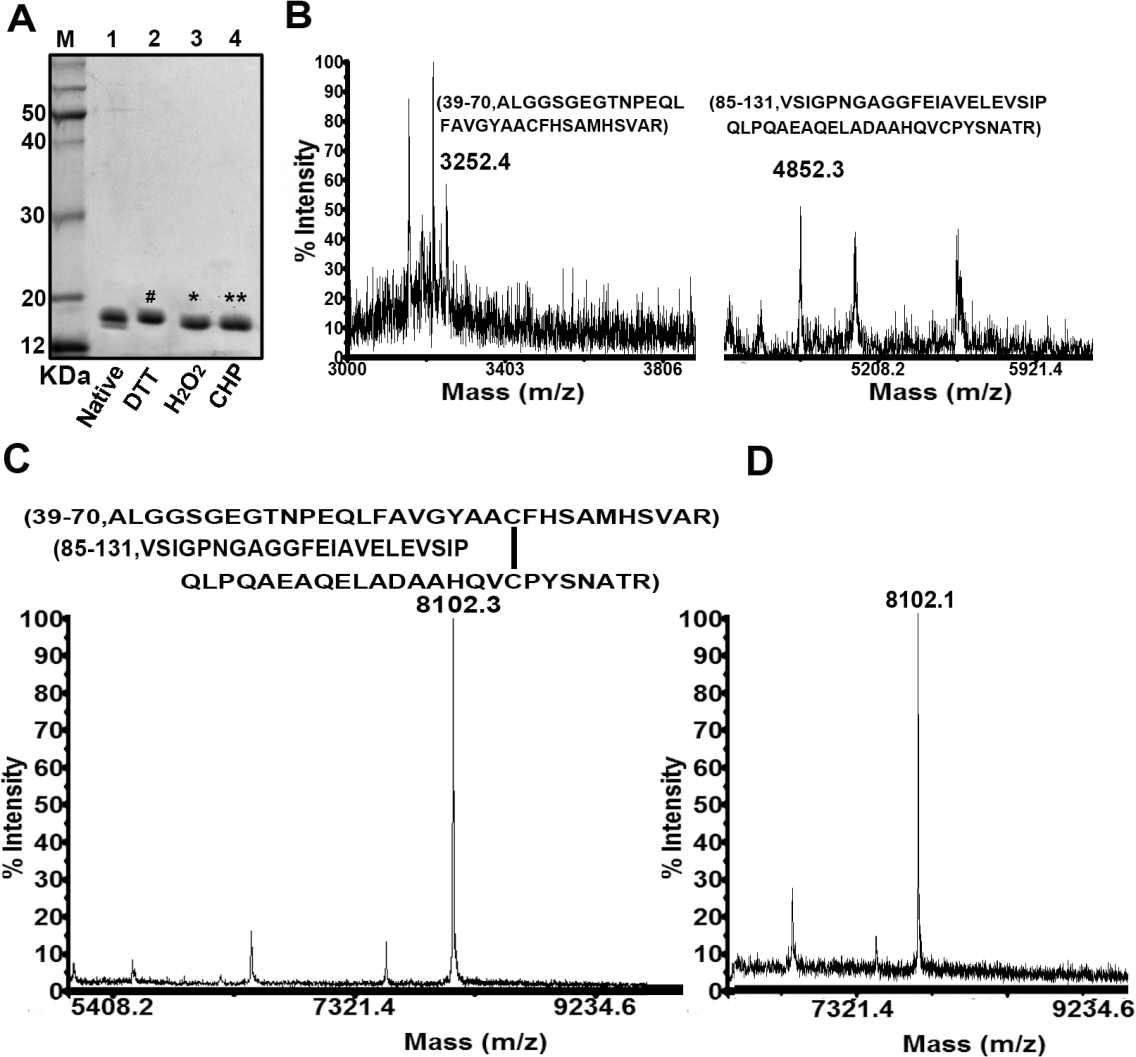
Intramolecular disulfide bond formation in Ohr. (**A**) Purified recombinant Ohr (Lane 1, control) was treated with 1 mM DTT, H_2_O_2,_ and CHP, respectively (Lanes 2–4), for 30 min at room temperature, and the proteins resolved by nonreducing 15% (w/v) SDS-PAGE. (**B**-**D**) MALDI-TOF MS-MS reveals disulfide bond formation. Bands (labeled ^#^ [**B**], * [**C**] and ** [**D**]) excised from the nonreducing gels in panel **A** were treated with trypsin and subjected to MS analysis. Only the relevant portion of each mass spectrum is shown.

To explore the biochemical activities of *C*. *glutamicum* Ohr, a recombinant protein was expressed in *E*. *coli* BL21 as an N-terminal His_6_-tagged fusion protein. The purified recombinant displayed as two bands on SDS-PAGE, both of which were approximately 17 kDa in size (**Fig 2A**, lane 1), consistent with the theoretical molecular mass. We hypothesized that the upper band might correspond to native Ohr protein and the lower to oxidized Ohr produced during purification. We confirmed this to be the case by showing that Ohr treated with H_2_O_2_ (**Fig 2A**, lane 3) and CHP (**Fig 2A**, lane 4) migrated to the position of the lower band, which was completely absent after treatment with DTT (**Fig 2A**, lane 2). MALDI-TOF MS-MS analysis further confirmed this hypothesis (**Fig 2B–2D**). The presence of 3,252.4-Da and 4,852.3-Da peaks, with molecular masses of the 39-to-70 peptide (ALGGSGEGTNPEQLFAVGYAAC^60^FH MHSVAR) and the 85-to-131 peptide (VSIGPNGAGGFEIAVELEVSIPQLPQAEAQELADAAHQVC^124^ PYSNATRGNIS), respectively, indicated that both Cys^60^ and Cys^124^ were in the thiol state in DTT-treated Ohr (**Fig 2B**). These two peaks were absent with oxidized Ohr. Also, a novel peak of 8,102.3 Da (**Fig 2C**), thus 2.4 Da smaller in size than the sum of the sizes of the 39-to-70 peptide (3,252.4 Da) and the 85-to-131 peptide (4,852.3 Da), was observed with both H_2_O_2_- (**Fig 2C**) and CHP-treated Ohr (**Fig 2D**), indicating formation of an intramolecular disulfide bond between Cys^60^ and Cys^124^. This rendered Ohr more compact, thus associating with a faster migration rate on nonreducing SDS-PAGE gels (**Fig 2A**).

**Fig 3:**
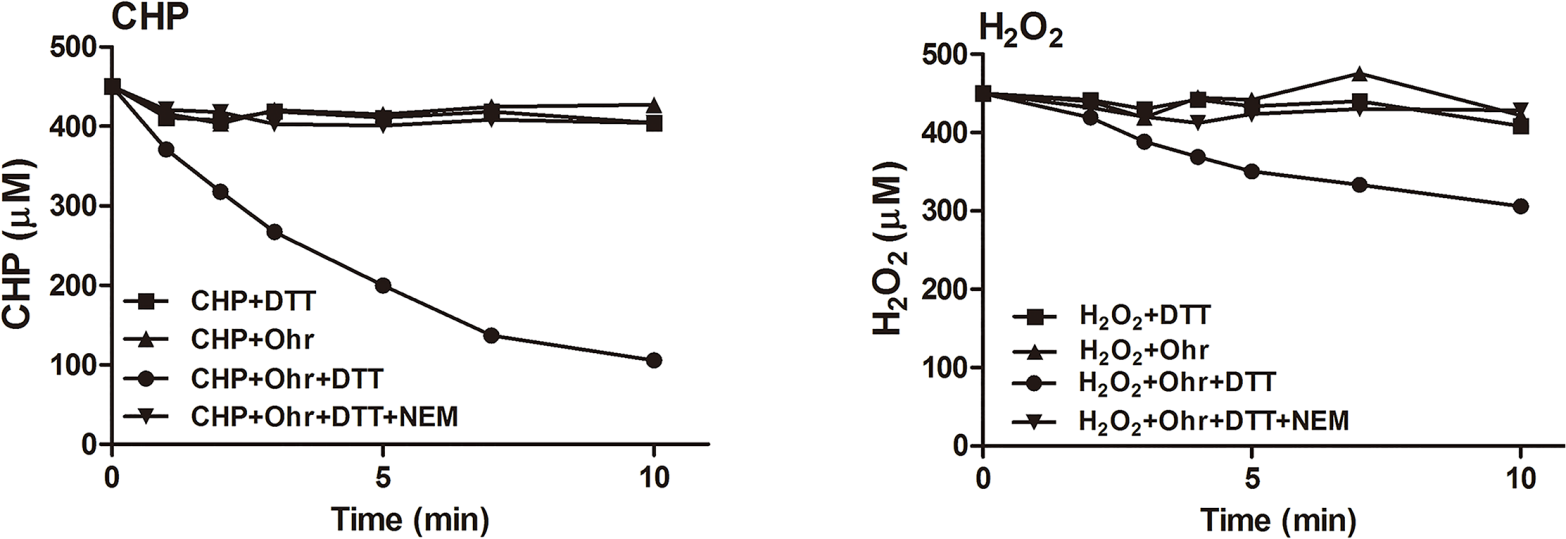
Peroxide-decomposition activity of Ohr. Peroxide concentrations were determined at the indicated time points using the FOX assay described in Material and Methods. **A:** The kinetics of CHP decomposition in the presence of Ohr (10 ng/μl). **B:** The kinetics of H_2_O_2_ decomposition in the presence of Ohr (50 ng/μl). All values are averages of data from three independent experiments. ●, full system (DTT + peroxide + Ohr); ▲, without DTT (Ohr + peroxide); ■, without Ohr (DTT+ peroxide); ▼, full system but Ohr was treated with NEM.

We next explored whether Ohr decomposes peroxides (**Fig 3**). Each reaction was initiated by addition of DTT; Ohr attacked peroxides only in the presence of DTT [12]. The Ohr-specific activities were 7.9 and 0.62 μm/min/ng, respectively, when CHP and H_2_O_2_ served as substrates. Thus, Ohr was approximately 12-fold more active against CHP than H_2_O_2_. The peroxidase activity was strongly inhibited by pretreatment with *N*-ethylmaleimide (NEM) for 1 h, indicating that the conserved Cys residues Cys^60^ and Cys^124^ are essential for peroxide decomposition.

### Ohr peroxidase activity requires the Cys residues

**Fig 4:**
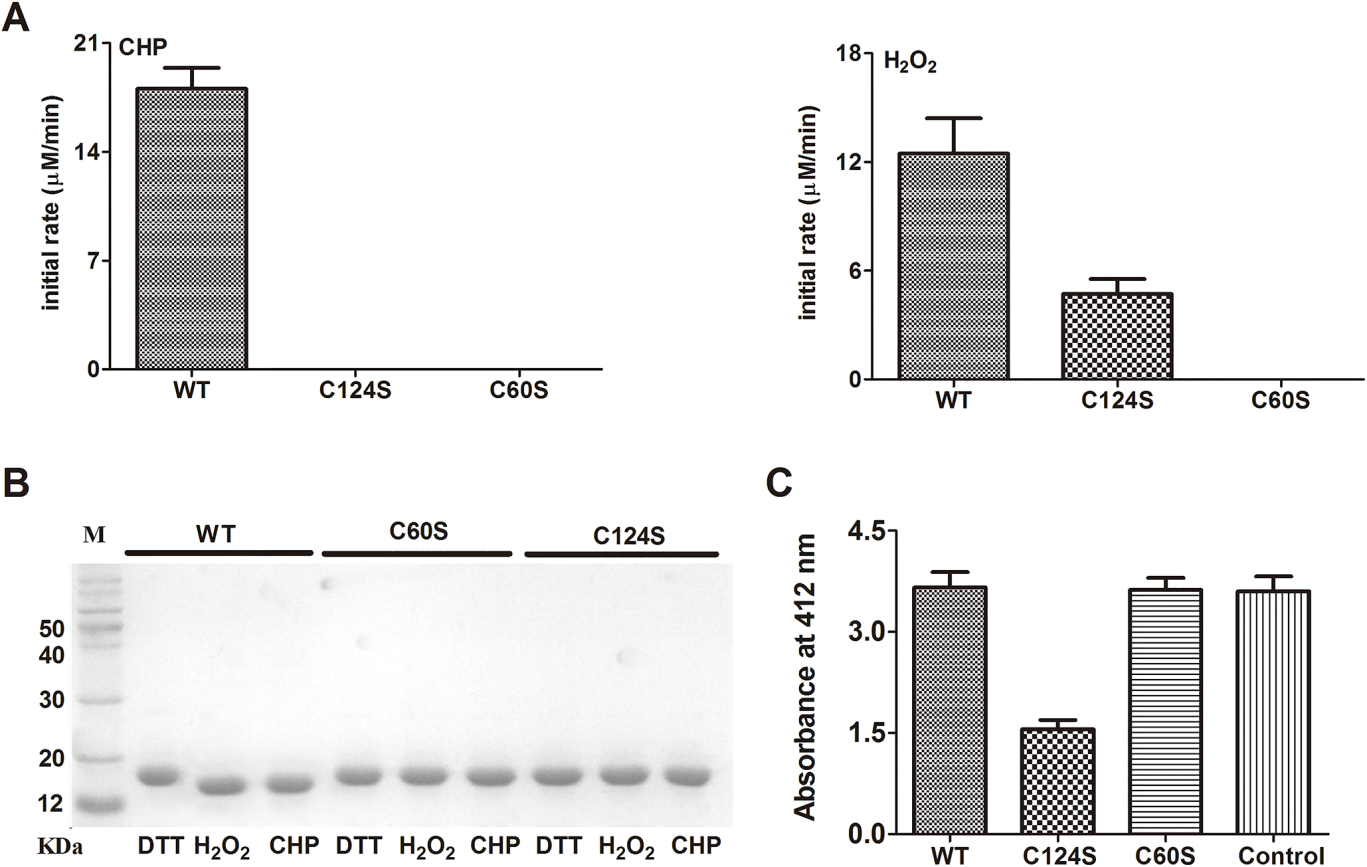
Effects of Cys residue removal on Ohr activity. (**A**) The initial rates of peroxide decomposition by Ohr, C124S, and C60S were measured using the FOX assay. The initial peroxide concentration was 200 μM, and all reactions were initiated by addition of DTT (0.5 mM). (**B**) Nonreducing 15% (w/v) SDS-PAGE analysis of wild-type Ohr and the C60S and C124S mutants treated with DTT, H_2_O_2_, and CHP (10 mM) for 30 min, respectively. (**C**) Sulfenic acid levels in the wild-type and mutant strains were measured after reaction with TNB. Mixed disulfides of Ohr, C124S, and C60S were prepared as described in Materials and Methods.

To further explore the roles played by the two Cys residues, we constructed Cys^60^ and Cys^124^ variants. As shown in **Fig 4A**, the activity against CHP was completely abolished in the C60S and C124S strains. However, when H_2_O_2_ served as the substrate, the C124S variant exhibited some peroxidase activity, but the C60S variant did not. Thus, both Cys residues play essential roles in CHP decomposition, and Cys^60^ is also critical in terms of H_2_O_2_ decomposition.

Both Cys^60^ and Cys^124^ are essential for intramolecular disulfide bond formation, as H_2_O_2_- and CHP- treated C60S and C124S variants migrated at the same position of DTT-treated wild-type protein, but slower than the oxidized wild-type protein, on nonreducing SDS-PAGE gels (**Fig 4B**). To identify the peroxidatic (C_P_) Cys, TNB [2-nitro-5-thiobenzoic acid] was used to identify sulfenic acid intermediates (R-SOH) in wild-type, C60S, and C124S Ohr. As shown in **Fig 4C**, the C124S protein formed a sulfenic acid intermediate, but neither the wild-type nor C60S variant did so, indicating that Cys^60^ is the peroxidatic Cys and is thus more reactive than Cys^124^ (**Fig 4C**). These data suggest that, during catalysis, Cys^60^ first reacts with a peroxide with concomitant formation of a sulfenic acid intermediate, which is then attacked by Cys^124^, triggering formation of an intramolecular disulfide bond between Cys^60^ and Cys^124^.

### The LpdA, Lpd/SucB and Trx/TrxR systems support the peroxidase activity of *C*. *glutamicum* Ohr

As reported earlier and confirmed in the present study, Ohr-mediated reduction of organic peroxides to less toxic organic alcohols is associated with formation of oxidized Ohr containing an intramolecular disulfide bond [12]. To complete the catalytic cycle *in vitro*, various reducing agents can be used to regenerate the sulfhydryl groups at the active Cys sites. Recently, Ohr regeneration *in vivo* (in *X*. *fastidiosa* [10] and *M*. *smegmatis* [13]) was shown to be mediated by a dedicated reducing system featuring lipoylated proteins (Lpd and SucB). Thus, we explored whether a lipoyl-dependent reducing system supports Ohr regeneration in *C*. *glutamicum*. Also, we determined whether the classical reducing systems of *C*. *glutamicum*, thus the Trx/TrxR and Mrx1/Mtr/MSH systems, support the peroxidase activity of Ohr. The Mrx1/Mtr/MSH reducing system is unique to MSH-producing high-G+C Gram-positive *Actinobacteria*, being functionally equivalent to the widespread Grx/GR/GSH system of most Gram-negative bacteria [25, 26]. The catalytic constants of Ohr in the presence of Trx, Mrx1, or LpdA and Lpd/SucB as recycling reductants were determined under steady-state conditions at saturating concentrations of peroxides (1 mM) and different concentrations of the reductants (0–120 μM). As shown in **Table 1**, the *k*_*cat*_ values of Ohr-mediated CHP reduction in the presence of the Trx, LpdA, and Lpd/SucB systems were 2.32±0.10 s^-1^, 5.31±0.03 s^-1^, and 4.72±0.19 s^-1^, respectively; and the respective *K*_m_ values were 24.51±0.83 μM, 12.13±0.51 μM, and 4.65±0.37 μΜ, respectively. The catalytic efficiencies were thus 9.50±0.74×10^4^ M^-1^ s^-1^, 43.83±1.57×10^4^ M^-1^ s^-1^, and 100.89±3.99×10^4^ M^-1^ s^-1^, respectively. However, all three reducing systems facilitated Ohr activity only poorly when H_2_O_2_ was used as the substrate. Thus, the LpdA and Lpd/SucB systems more effectively support Ohr peroxidase activity than does the Trx system when CHP is the substrate; the Lpd/SucB system had the highest regenerative activity. Under all conditions evaluated, the Mrx1/Mtr/MSH reducing system failed to support Ohr activity when either CHP or H_2_O_2_ was the substrate (**Table 1**). Ohr activities were also measured in the presence of fixed concentrations (40 μM) of the Trx, LpdA, and Lpd/SucB reducing systems and different concentrations of peroxides (0–1 mM) (**Table 2**). As expected, Ohr reduced CHP at least 10,000-fold more efficiently than H_2_O_2_ when LpdA and Lpd/SucB provided the reducing power, and at least 1,700-fold more efficiently when Trx served to that end. Together, the data suggest that Ohr can be regenerated by both the lipoyl-dependent and the classic Trx/TrxR reducing systems of *C*. *glutamicum*, but the lipoylated proteins (LpdA and Lpd/SucB) are the prime electron donors.

**Table 1:** Kinetic constants of different Ohr reducing systems.

Systems	Substrates	*K*_*m*_ (μM)	*k*_*cat*_ (s^-1^)	*k*_*cat*_/*K*_*m*_ (×10^4^ M^-1^s^-1^)
Lpd/SucB	CHP	4.65±0.37	4.72±0.19	100.89±3.99
	H_2_O_2_	25.79±0.66	2.39±0.02	9.25±0.18
LpdA	CHP	12.13±0.51	5.31±0.03	43.83±1.57
	H_2_O_2_	22.97±0.09	2.33±0.03	10.13±0.06
Trx/TrxR	CHP	24.51±0.83	2.32±0.10	9.50±0.74
	H_2_O_2_	33.85±0.44	2.76±0.01	8.17±0.15
Mrx1/Mtr/MSH	CHP	ND	ND	ND
	H_2_O_2_	ND	ND	ND

Peroxidase assays were performed as described in Materials and Methods using fixed concentrations of peroxides (1 mM) and Ohr (0.1 μM) and different concentrations of reducing systems: the LpdA system (0–120 μM LpdA), Lpd/SucB system (0–120 μM Lpd and 5 μM SucB), Trx system (0–120 μM Trx and 5 μM TrxR), and the Mrx1 system (0–120 μM Mrx1, 5 μM Mtr and 500 μM MSH). All data represent the means obtained from three independent assays. ND, not detectable.

**Table 2:** Kinetic constants for different Ohr substrates.

Substrate	System	*K*_*m*_ (μM)	*k*_*cat*_ (s^-1^)	*k*_*cat*_/*K*_*m*_ (×10^3^ M^-1^s^-1^)
CHP	Lpd/SucB	17.48±0.78	5.48±0.00	314.32±1.42
	LpdA	21.75±0.98	3.53±0.09	162.61±3.22
	Trx/TrxR	296.60±10.04	2.74±0.61	9.17±1.76
H_2_O_2_	Lpd/SucB	708.80±23.94	0.92±0.14	1.29±0.16
	LpdA	843.01±39.17	1.50±0.08	1.77±0.02
	Trx/TrxR	948.00±25.55	4.93±0.08	5.21±0.05

Peroxidase assays were performed as described in Materials and Methods with the concentrations of all components except the peroxides (eight concentrations; 0–1 mM) held constant. To determine Michaelis constants, all reaction mixtures contained 50 mM sodium phosphate (pH 7.4), 50 mM NaCl, 1 mM DTPA (pH 7.4), 0.2 mM NADPH, 0.1 μM Ohr, and either the Lpd/SucB system (5 μM SucB and 40 μM Lpd), the LpdA system (40 μM), or the Trx system (5 μM TrxR and 40 μM Trx). The data represent the means obtained from three independent assays.

### Survival of the *ohr* mutant is compromised by organic peroxides

**Fig 5:**
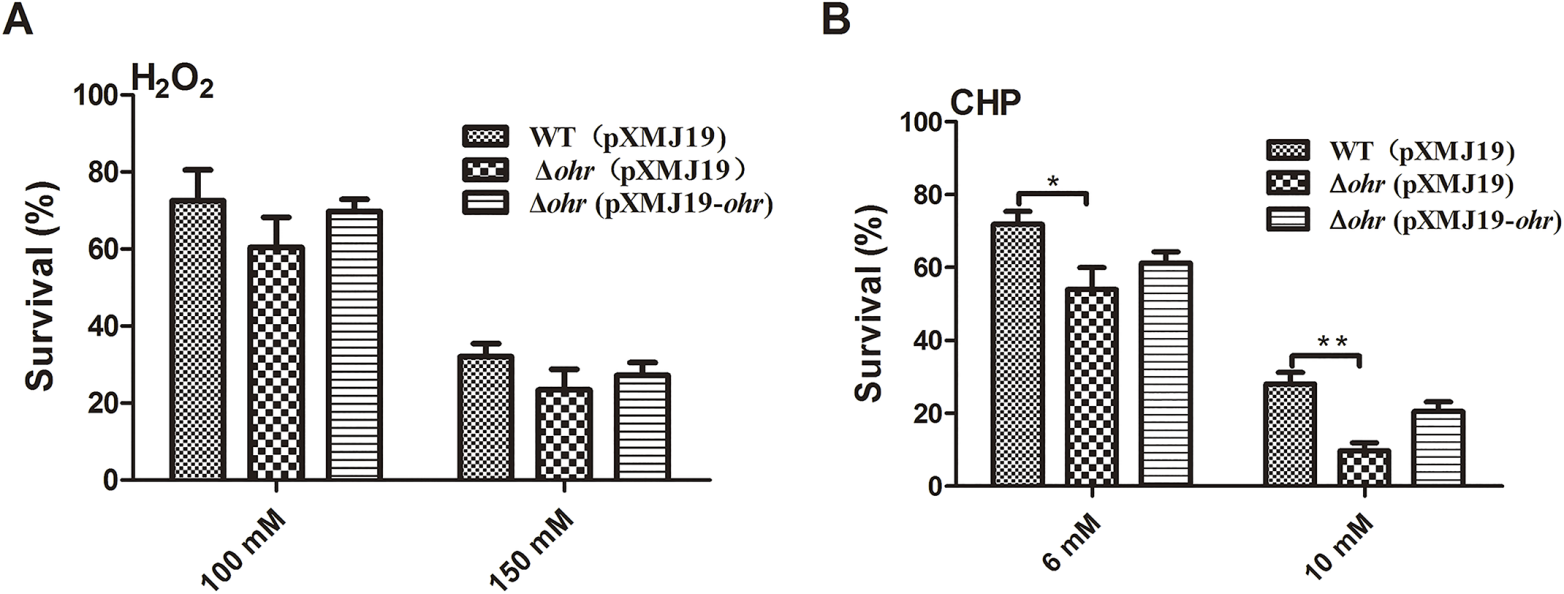
Effects of Ohr on *C*. *glutamicum* resistance to CHP and H_2_O_2_. The survival levels of *C*. *glutamicum* WT (pXMJ19), Δ*ohr*(pXMJ19), and Δ*ohr*(pXMJ19-*ohr*) after exposure to the stressors (**A**) H_2_O_2_ (100 and 150 mM) and (**B**) CHP (6 and 10 mM) for 30 min, respectively. Mean values ± standard deviations (error bars) from three replicate assays are shown. **: *P*≤0.01.*: *P*≤0.05.

Ohr from other bacterial species plays an important role in the decomposition of organic hydroperoxides [5, 11, 17]. Therefore, we speculated that Ohr from *C*. *glutamicum* has the same function. To confirm a role of Ohr in mediating resistance to oxidative stress, we constructed a Δ*ohr* deletion mutant and assessed the sensitivity thereof to CHP and H_2_O_2_ (**Fig 5**). The Δ*ohr* mutant was not significantly less resistant to H_2_O_2_ than was the wild-type strain at even a high H_2_O_2_ concentration (150 mM) (**Fig 5A**). However, upon addition of 6 mM CHP, survival of the Δ*ohr* mutant was only approximately 70% that of the wild-type, and this decreased further to 25% with 10 mM CHP (**Fig 5B**). Moreover, the hypersensitivity of the Δ*ohr* mutant to both H_2_O_2_ and CHP was partially restored when the genomic mutation was complemented by the wild-type gene of plasmid pXMJ19-*ohr* **(Fig 5)**. These results show that the Ohr of *C*. *glutamicum* plays an important role in the defense against organic peroxides, as does Ohr of other bacteria [5, 11, 36].

### *C*. *glutamicum* Ohr reduces intracellular ROS levels *in vivo*

**Fig 6:**
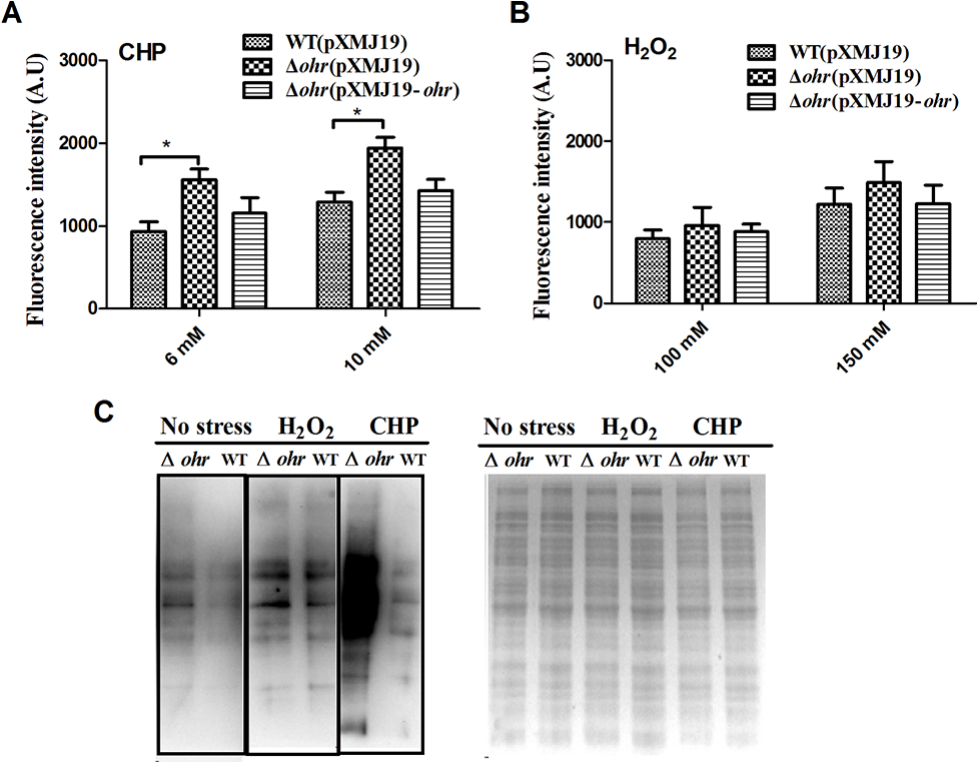
Ohr reduces ROS production and protein oxidation in cells exposed to CHP and H_2_O_2_. The intracellular ROS levels of the wild-type (pXMJ19), Δ*ohr*(pXMJ19), and Δ*ohr*(pXMJ19-*ohr*) strains were measured using the DCFH-DA fluorescence assay after exposure of the cells to the indicated concentrations of CHP (**A**) and H_2_O_2_ (**B**) for 30 min at 30°C. The bars represent fluorescence intensities in arbitrary units (A.U.). Mean values ± standard deviations (error bars) from three replicate experiments are shown. *: *P*≤0.05. (**C**) The protein carbonyl contents of *C*. *glutamicum* wild-type (lanes 2, 4, and 6) and the Δ*ohr* mutant (lanes 1, 3, and 5) in cells exposed to H_2_O_2_ (150 mM) or CHP (10 mM) for 30 min at 30°C were analyzed via Western blotting using an anti-dinitrophenyl antibody. The side panel shows Coomassie Brilliant Blue-stained proteins.

Of the various types of ROS, organic peroxides are particularly toxic, partly because these compounds engage in free radical reactions generating reactive organic radicals that in turn damage membranes and other macromolecules [2]. To explore the physiological role played by Ohr in removal of ROS when *C*. *glutamicum* is oxidatively stressed, intracellular ROS levels were measured using the fluorogenic probe 2’,7’-dichlorodihydrofluorescein diacetate. As shown in **Fig 6A**, the wild-type strain had a significantly lower ROS level than that of the Δ*ohr* mutant after exposure to CHP for 30 min. However, the ROS level in the Δ*ohr* mutant was partially restored to that of the wild-type by introducing the complementation plasmid pXMJ19-*ohr* (**Fig 6A**). The ROS levels of Δ*ohr* mutant also increased when treated with H_2_O_2_ (100 mM and 150 mM) compared to the wild-type, although the increase is not as strong as induced by CHP treatment (**Fig 6B**). These findings suggest that *C*. *glutamicum* Ohr inhibits intracellular ROS accumulation triggered by peroxides.

Cys thiol groups are especially susceptible to modification by ROS that elude antioxidant defense systems, causing irreversible formation of methionine sulfoxide and Cys disulfide bonds, eventually triggering protein carbonylation [37]. As Ohr inhibits intracellular ROS accumulation by *C*. *glutamicum*, we explored whether Ohr protects cells against protein carbonylation under conditions of oxidative stress. We prepared total protein suspensions of wild-type and Δ*ohr* mutant strains grown in the presence of CHP or H_2_O_2_. Protein carbonyl groups were derivatized with 2,4-dinitrophenyl hydrazine, and these derivatives were detected via Western blotting using an anti-dinitrophenyl antibody. As expected, the extent of protein carbonylation in the wild-type was significantly lower than that of the Δ*ohr* mutant after growth under CHP stress. However, this was not the case with H_2_O_2_ stress **(Fig 6C**). Together, the data indicate that Ohr plays an important role in protection against oxidative stress induced by organic hydroperoxides; Ohr inhibits intracellular ROS accumulation and protein carbonylation.

### CHP induces Ohr expression

**Fig 7:**
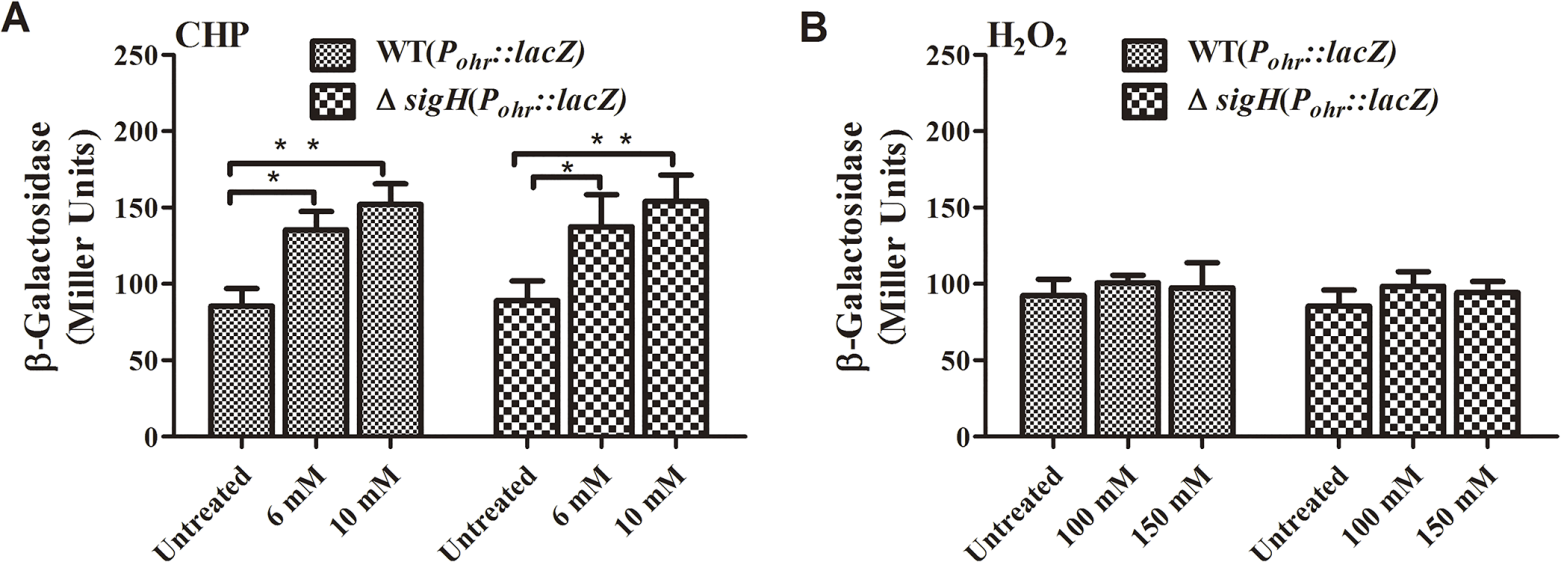
Induction of *ohr* by CHP and H_2_O_2_. The expression levels of *ohr* in the presence of CHP (**A**) or H_2_O_2_ (**B**) in *C*. *glutamicum* wild-type and Δ*sigH* mutant strains containing a *P*_*ohr*_::*lacZ* chromosomal fusion reporter. The β-galactosidase assay was used to measure promoter activities. Error bars represent the standard deviations (SD) from three replicate tests. **: *P*≤0.01.*: *P*≤0.05.

As Ohr promoted *C*. *glutamicum* survival under conditions of oxidative stress, we measured *ohr* expression in the presence of CHP or H_2_O_2_ using a chromosomal *P*_*ohr*_::*lac*Z fusion reporter. As shown in **Fig 7A**, the wild-type *P*_*ohr*_ promoter activity increased by 58% and 77% upon exposure to 6 mM and 10 mM CHP, respectively, compared with the untreated control. However, H_2_O_2_ did not obviously induce *ohr* expression (**Fig 7B**). These data show that the organic peroxide CHP specifically induced *ohr* expression, directly contributing to CHP tolerance.

Such specific induction of *ohr* by CHP suggests that a transcriptional regulator might be at play. In many bacteria, *ohr* expression is regulated by OhrR, a transcriptional repressor of the MarR family [34, 38]. However, no *ohrR* homolog is evident in the *C*. *glutamicum* genome. Recently, several studies have found that the stress-responsive extracytoplasmic function-sigma (ECF-σ) factor SigH regulates the expression of many oxidative stress resistance genes in *C*. *glutamicum* [39, 40]. We thus measured *ohr-lacZ* expression in a Δ*sigH* mutant. However, *ohr* expression did not obviously differ between the Δ*sigH* mutant and the wild-type strain exposed to either CHP or H_2_O_2_ (**Fig 7**). Thus, *ohr* expression in *C*. *glutamicum* appears to be regulated by an unknown mechanism that responds specifically to organic peroxides.

## Discussion

Our results show that *ohr* plays an essential role in organic hydroperoxide reduction in *C*. *glutamicum*. *Ohr* deletion significantly increased bacterial sensitivity to the organic hydroperoxide CHP, and such sensitivity was nearly restored to wild-type levels upon complementation with the *ohr* gene. The Δ*ohr* mutant was clearly more sensitive to CHP than to H_2_O_2_. Also, intracellular ROS accumulation and the associated protein carbonylation were significantly higher (compared with wild-type) in the Δ*ohr* mutant exposed to CHP, but only slight increases were evident upon exposure to H_2_O_2_, suggesting that Ohr plays a vital role in the decomposition of organic hydroperoxides. These results are in line with the roles played by Ohr in other organisms, specifically in imparting resistance to organic peroxides [5, 11].

*C*. *glutamicum* Ohr contains two conserved Cys residues. Cys^60^, located within a very hydrophobic environment, is directly involved in peroxide reduction, whereas Cys^124^ (located in a hydrophilic environment) is the resolving Cys [12]. During catalysis, Cys^60^ first reacts with a peroxide, with concomitant formation of a sulfenic acid intermediate, which is then attacked by Cys^124^, leading to formation of an intramolecular disulfide bond between Cys^60^ and Cys^124^ and release of a molecule of water. The disulfide bond is directly reduced by either the Trx/TrxR or the lipoyl-dependent reducing system to complete the catalytic cycle.

Recently, Ohr of *X*. *fastidiosa* [10] and *M*. *smegmatis* [13] was shown to be regenerated by a special cellular reducing system featuring lipoylated proteins (Lpd and SucB). In the present study, we confirmed that oxidized *C*. *glutamicum* Ohr is also efficiently reduced by LpdA- and Lpd/SucB-coupled systems, suggesting that lipoylated protein-based reducing systems reduce the Ohr enzymes of various bacteria. Indeed, the Lpd and SucB reducing systems also serve as electron donors for OsmC and AhpC [10, 41]. To further explore reducing systems donating electrons to Ohr, we determined whether the classical Trx/TrxR and Mrx1/Mtr/MSH systems supported Ohr peroxidase activity. Although earlier work indicated that neither Trx nor Grx reduced the Ohr of *X*. *fastidiosa*, it is important to note that this bacterium has at least three Grx- and four Trx-encoding genes. Therefore, the possibility that (an) alternative Trx and/or Grx donate(s) electrons to Ohr cannot be excluded. In fact, the Trx/TrxR system functioned weakly, but not negligibly, to reduce *C*. *glutamicum* Ohr; the LpdA and Lpd/SucB systems were much more efficient. The Mrx/Mtr/MSH system did not support the Ohr activity of *M*. *smegmatis* [13]. We showed here that this was also true of *C*. *glutamicum* (**Table 2**).

Traditionally, Ohr is regulated by OhrR, a transcriptional repressor of the MarR family [42]. However, we could not identify an OhrR homolog in *C*. *glutamicum*, suggesting that *ohr* is regulated by a different mechanism. In *B*. *subtilis*, OhrR mutation eliminates regulation of *ohrA*, but not *ohrB*. Indeed, *ohrB* expression is regulated in a σ^B^-dependent manner, suggesting that *ohr* can also be regulated by sigma factors [34]. Recently, several studies found that the stress-responsive ECF-σ factor SigH regulates the expression of many oxidative stress resistance genes in *C*. *glutamicum* [39, 40]. Interestingly, both the basal and inducible expression of *sigB* in *C*. *glutamicum* was reported to be regulated by SigH [43]. These findings prompted us to explore whether Ohr of *C*. *glutamicum* was also regulated by SigH. However, we found no obvious difference in *ohr* expression levels between the Δ*sigH* mutant and the wild-type strain exposed to either CHP or H_2_O_2_. Thus, *ohr* expression in *C*. *glutamicum* may be regulated by an unknown mechanism responding specifically to organic hydroperoxides.

In conclusion, we found that *C*. *glutamicum* Ohr was essential for conferring effective resistance to a highly toxic organic hydroperoxide, which it decomposed more efficiently than did hydrogen peroxide. These results improve our knowledge of stress resistance in *C*. *glutamicum* and indicate how future robust industrial strains may be engineered.

## Supporting Information

S1 Table Bacterial strains and plasmids used in this study. (DOCX)

S2 Table Primers used in this study. (DOCX)

## References

[1] Chandra J, Samali A, Orrenius S. Triggering and modulation of apoptosis by oxidative stress. Free Radical Biol Med. 2000;29(3):323–33. 11035261 10.1016/s0891-5849(00)00302-6

[2] Halliwell B, Gutteridge J. Oxygen toxicity, oxygen radicals, transition metals and disease. Biochem J. 1984;219(1):1 6326753 10.1042/bj2190001 PMC1153442

[3] Baker CJ, Orlandi EW. Active oxygen in plant pathogenesis. Annu Rev Phytopathol. 1995;33(1):299–321. 18999963 10.1146/annurev.py.33.090195.001503

[4] Akaike T, Sato K, Ijiri S, Miyamoto Y, Kohno M, Ando M, et al Bactericidal activity of alkyl peroxyl radicals generated by heme-iron-catalyzed decomposition of organic peroxides. Arch Biochem Biophys. 1992;294(1):55–63. 1312811 10.1016/0003-9861(92)90136-k

[5] Mongkolsuk S, Praituan W, Loprasert S, Fuangthong M, Chamnongpol S. Identification and characterization of a new organic hydroperoxide resistance (*ohr*) gene with a novel pattern of oxidative stress regulation from *Xanthomonas campestrispv*. *phaseoli* . J Bacteriol. 1998;180(10):2636–43. 9573147 10.1128/jb.180.10.2636-2643.1998 PMC107214

[6] Wang G, Alamuri P, Maier RJ. The diverse antioxidant systems of *Helicobacter pylori* . Mol Microbiol. 2006;61(4):847–60. 16879643 10.1111/j.1365-2958.2006.05302.x

[7] Lesniak J, Barton WA, Nikolov DB. Structural and functional characterization of the *Pseudomonas* hydroperoxide resistance protein Ohr. EMBO J. 2002;21(24):6649–59. 12485986 10.1093/emboj/cdf670 PMC139091

[8] Atichartpongkul S, Loprasert S, Vattanaviboon P, Whangsuk W, Helmann JD, Mongkolsuk S. Bacterial Ohr and OsmC paralogues define two protein families with distinct functions and patterns of expression. Microbiology. 2001;147(7):1775–82. 11429455 10.1099/00221287-147-7-1775

[9] Lesniak J, Barton WA, Nikolov DB. Structural and functional features of the *Escherichia coli* hydroperoxide resistance protein OsmC. Protein Sci. 2003;12(12):2838–43. 14627744 10.1110/ps.03375603 PMC2366992

[10] Cussiol JRR, Alegria TGP, Szweda LI, Netto LES. Ohr (Organic Hydroperoxide Resistance Protein) possesses a previously undescribed activity, lipoyl-dependent peroxidase. J Biol Chem. 2010;285(29):21943–50. 10.1074/jbc.M110.117283 20463026 PMC2903379

[11] Ochsner UA, Hassett DJ, Vasil ML. Genetic and physiological characterization of *ohr*, encoding a protein involved in organic hydroperoxide resistance in *Pseudomonas aeruginosa* . J Bacteriol. 2001;183(2):773–8. 11133975 10.1128/JB.183.2.773-778.2001 PMC94937

[12] Cussiol JR, Alves SV, de Oliveira MA, Netto LE. Organic hydroperoxide resistance gene encodes a thiol-dependent peroxidase. J Biol Chem. 2003;278(13):11570–8. 12540833 10.1074/jbc.M300252200

[13] Ta P, Buchmeier N, Newton GL, Rawat M, Fahey RC. Organic hydroperoxide resistance protein and ergothioneine compensate for loss of mycothiol in *Mycobacterium smegmatis* mutants. J Bacteriol. 2011;193(8):1981–90. 10.1128/JB.01402-10 21335456 PMC3133051

[14] Caswell CC, Baumgartner JE, Martin DW, Roop RM. Characterization of the organic hydroperoxide resistance system of *Brucella abortus* 2308. J Bacteriol. 2012;194(18):5065–72. 10.1128/JB.00873-12 22821968 PMC3430311

[15] Chuchue T, Tanboon W, Prapagdee B, Dubbs JM, Vattanaviboon P, Mongkolsuk S. *ohr*R and *ohr* are the primary sensor/regulator and protective genes against organic hydroperoxide stress in *Agrobacterium tumefaciens* . J Bacteriol. 2006;188(3):842–51. 16428387 10.1128/JB.188.3.842-851.2006 PMC1347339

[16] Jenkins C, Samudrala R, Geary SJ, Djordjevic SP. Structural and functional characterization of an organic hydroperoxide resistance protein from *Mycoplasma gallisepticum* . J Bacteriol. 2008;190(6):2206–16. 10.1128/JB.01685-07 18192392 PMC2258871

[17] Shea RJ, Mulks MH. *ohr*, encoding an organic hydroperoxide reductase, is an *in vivo*-induced gene in *Actinobacillus pleuropneumoniae* . Infect Immun. 2002;70(2):794–802. 11796613 10.1128/iai.70.2.794-802.2002 PMC127688

[18] de Abreu Meireles D, Alegria TGP, Alves SV, Arantes CRR, Netto LES. A 14.7 kDa protein from *Francisella tularensis* subsp. novicida (Named FTN_1133), involved in the response to oxidative stress induced by organic peroxides, is not endowed with thiol-dependent peroxidase activity. PLoS one. 2014;9(6):e99492 10.1371/journal.pone.0099492 24959833 PMC4069020

[19] Bröer S, Eggeling L, Krämer R. Strains of *Corynebacterium glutamicum* with different lysine productivities may have different lysine excretion systems. Appl Environ Microbiol. 1993;59(1):316–21. 16348855 10.1128/aem.59.1.316-321.1993 PMC202097

[20] Lee J-Y, Seo J, Kim E-S, Lee H-S, Kim P. Adaptive evolution of *Corynebacterium glutamicum* resistant to oxidative stress and its global gene expression profiling. Biotechnol Lett. 2013;35(5):709–17. 10.1007/s10529-012-1135-9 23288296

[21] Liu Y-B, Long M-X, Yin Y-J, Si M-R, Zhang L, Lu Z-Q, et al Physiological roles of mycothiol in detoxification and tolerance to multiple poisonous chemicals in *Corynebacterium glutamicum* . Arch Microbiol. 2013;195(6):419–29. 10.1007/s00203-013-0889-3 23615850

[22] Bott M, Niebisch A. The respiratory chain of *Corynebacterium glutamicum* . J Biotechnol. 2003;104(1):129–53. 12948635 10.1016/s0168-1656(03)00144-5

[23] El Shafey H, Ghanem S, Merkamm M, Guyonvarch A. *Corynebacterium glutamicum* superoxide dismutase is a manganese-strict non-cambialistic enzyme *in vitro* . Microbiol Res. 2008;163(1):80–6. 16809027 10.1016/j.micres.2006.05.005

[24] Teramoto H, Inui M, Yukawa H. OxyR acts as a transcriptional repressor of hydrogen peroxide‐inducible antioxidant genes in *Corynebacterium glutamicum* R. FEBS J. 2013;280(14):3298–312. 10.1111/febs.12312 23621709

[25] Si M-R, Zhang L, Yang Z-F, Xu Y-X, Liu Y-B, Jiang C-Y, et al NrdH redoxin enhances resistance to multiple oxidative stresses by acting as a peroxidase cofactor in *Corynebacterium glutamicum* . Appl Environ Microbiol. 2014;80(5):1750–62. 10.1128/AEM.03654-13 24375145 PMC3957609

[26] Ordóñez E, Van Belle K, Roos G, De Galan S, Letek M, Gil JA, et al Arsenate reductase, mycothiol, and mycoredoxin concert thiol/disulfide exchange. J Biol Chem. 2009;284(22):15107–16. 10.1074/jbc.M900877200 19286650 PMC2685692

[27] Zhang W, Wang Y, Song Y, Wang T, Xu S, Peng Z, et al A type VI secretion system regulated by OmpR in *Yersinia pseudotuberculosis* functions to maintain intracellular pH homeostasis. Environ Microbiol. 2013;15(2):557–69. 10.1111/1462-2920.12005 23094603

[28] Shen X-H, Jiang C-Y, Huang Y, Liu Z-P, Liu S-J. Functional identification of novel genes involved in the glutathione-independent gentisate pathway in *Corynebacterium glutamicum* . Appl Environ Microbiol. 2005;71(7):3442–52. 16000747 10.1128/AEM.71.7.3442-3452.2005 PMC1169049

[29] Jiang ZY, Hunt JV, Wolff SP. Ferrous ion oxidation in the presence of xylenol orange for detection of lipid hydroperoxide in low density lipoprotein. Anal Biochem. 1992;202(2):384–9. 1519766 10.1016/0003-2697(92)90122-n

[30] Ellis HR, Poole LB. Novel application of 7-chloro-4-nitrobenzo-2-oxa-1,3-diazole to identify cysteine sulfenic acid in the AhpC component of alkyl hydroperoxide reductase. Biochemistry. 1997;36(48):15013–8. 9398227 10.1021/bi972191x

[31] Schurig-Briccio L, Farías R, Rodríguez-Montelongo L, Rintoul M, Rapisarda V. Protection against oxidative stress in *Escherichia coli* stationary phase by a phosphate concentration-dependent genes expression. Arch Biochem Biophys. 2009;483(1):106 10.1016/j.abb.2008.12.009 19138658

[32] Vinckx T, Wei Q, Matthijs S, Noben J-P, Daniels R, Cornelis P. A proteome analysis of the response of a *Pseudomonas aeruginosa oxyR* mutant to iron limitation. Biometals. 2011;24(3):523–32. 10.1007/s10534-010-9403-4 21207115

[33] Miller J. A short course in bacterial genetics: a laboratory manual and handbook for *Escherichia coli* and related bacteria. Trends Biochem Sci. 1993;18:193.

[34] Fuangthong M, Atichartpongkul S, Mongkolsuk S, Helmann JD. OhrR is a repressor of *ohrA*, a key organic hydroperoxide resistance determinant in *Bacillus subtilis* . J Bacteriol. 2001;183(14):4134–41. 11418552 10.1128/JB.183.14.4134-4141.2001 PMC95301

[35] Oh S-Y, Shin J-H, Roe J-H. Dual role of OhrR as a repressor and an activator in response to organic hydroperoxides in *Streptomyces coelicolor* . J Bacteriol. 2007;189(17):6284–92. 17586628 10.1128/JB.00632-07 PMC1951921

[36] da Silva Neto JF, Negretto CC, Netto LE. Analysis of the organic hydroperoxide response of *Chromobacterium violaceum* reveals that OhrR is a Cys-based redox sensor regulated by thioredoxin. PLoS one. 2012;7(10):e47090 10.1371/journal.pone.0047090 23071722 PMC3469484

[37] Nyström T. Role of oxidative carbonylation in protein quality control and senescence. EMBO J. 2005;24(7):1311–7. 15775985 10.1038/sj.emboj.7600599 PMC1142534

[38] Sukchawalit R, Loprasert S, Atichartpongkul S, Mongkolsuk S. Complex regulation of the organic hydroperoxide resistance gene (*ohr*) from *Xanthomonas* involves OhrR, a novel organic peroxide-inducible negative regulator, and posttranscriptional modifications. J Bacteriol. 2001;183(15):4405–12. 11443074 10.1128/JB.183.15.4405-4412.2001 PMC95334

[39] Si M, Long M, Chaudhry MT, Xu Y, Zhang P, Zhang L, et al Functional characterization of *Corynebacterium glutamicum* mycothiol S-conjugate amidase. PLoS one. 2014;9(12):e115075 10.1371/journal.pone.0115075 25514023 PMC4267739

[40] Si M, Zhang L, Chaudhry MT, Ding W, Xu Y, Chen C, et al. *Corynebacterium glutamicum* methionine sulfoxide reductase A uses both mycoredoxin and thioredoxin for regeneration and oxidative stress resistance. Appl Environ Microbiol. 2015:AEM. 04221–14. 10.1128/AEM.04221-14 PMC4375309 25681179

[41] Bryk R, Lima CD, Erdjument-Bromage H, Tempst P, Nathan C. Metabolic enzymes of mycobacteria linked to antioxidant defense by a thioredoxin-like protein. Science. 2002;295(5557):1073–7. 11799204 10.1126/science.1067798

[42] Li N, Luo Q, Jiang Y, Wu G, Gao H. Managing oxidative stresses in *Shewanella oneidensis*: intertwined roles of the OxyR and OhrR regulons. Environ Microbiol. 2014;16(6): 1821–1834 25009841 10.1111/1462-2920.12418

[43] Ehira S, Teramoto H, Inui M, Yukawa H. Regulation of *Corynebacterium glutamicum* heat shock response by the extracytoplasmic-function Sigma factor SigH and transcriptional regulators HspR and HrcA. J Bacteriol. 2009;191(9):2964–72. 10.1128/JB.00112-09 19270092 PMC2681815

