# Supplementary material for: Ohr Protects Corynebacterium glutamicum against Organic Hydroperoxide Induced Oxidative Stress
Source: PLoS One. 2015 Jun 29;10(6):e0131634. doi: 10.1371/journal.pone.0131634 (PMC4486171; doi:10.1371/journal.pone.0131634)
Supplement: S1 Table — (DOCX) [file pone.0131634.s001.docx]

**Table S1. Bacterial strains and plasmids used in this study.**

| **Strains and plasmids** | **Relevant characteristics** | **Reference** |
| --- | --- | --- |
| **Strains** |  |  |
| ***E. coli*** |  |  |
| BL21(DE3) | Host for expression vector pET28a and pET15b | Novagen |
| JM109 | Host for cloning | Stratagene |
| ***C. glutamicum*** |  |  |
| RES167 | Restriction-deficient mutant of ATCC13032;  Δ(*cglIM-cglIR-cglIIR*) | [[1](#_ENREF_46)] |
| Δ*ohr* | *ohr* deleted in RES167 | This study |
| Δ*sigH* | *sigH* deleted in RES167 | [[2](#_ENREF_38)] |
| WT(pXMJ19) | RES167 containing pXMJ19 vector | This study |
| Δ*ohr*(pXMJ19-*ohr*) | Complementation of *ohr* in Δ*ohr* mutant | This study |
| **Plasmids** |  |  |
| pK18*mobsacB* | Suicide vector allows for selection of double-crossover in  *C. glutamicum* | [[3](#_ENREF_47)] |
| pK18*mobsacB-*Δ*ohr* | Construct used for in-frame deletion of the *ohr* gene | This study |
| pK18*mobsacB*-*P_ohr_::lacZ* | *P_ohr_::lacZ* promoter fusion in pK18*mobsacB* | This study |
| pXMJ19 | Shuttle vector (*Ptac lacI^q^ pBL1 oriV_C. glutamicum_* pK18 *oriV_E. coli_*) | [[4](#_ENREF_48)] |
| pXMJ19-*ohr* | *ohr* cloned into pXMJ19 for complementation | This study |
| pET28a | Expression vector with N-terminal hexahistidine affinity tag | Novagen |
| pET28a-*ohr* | pET28a derivative for expression of *ohr* (*ncgl0023*) | This study |
| pET28a-*ohr::C60S* | pET28a derivative for expression of *ohr::C60S* | This study |
| pET28a-*ohr::C124S* | pET28a derivative for expression of *ohr::C124S* | This study |
| pET28a-*lpdA* | pET28a derivative for expression of *lpdA* (*ncgl0658*) | This study |
| pET28a-*sucB* | pET28a derivative for expression of *sucB* (*ncgl2126*) | This study |
| pET28a-*mtr* | pET28a derivative for expression of *mtr* (*ncgl1928*) | [[5](#_ENREF_25)] |
| pET28a-*trx* | pET28a derivative for expression of *trx* (*ncgl2985*) | [[5](#_ENREF_25)] |
| pET28a-*trxR* | pET28a derivative for expression of *trxR* (*ncgl2984*) | [[5](#_ENREF_25)] |
| pET28a-*mrx1* | pET28a derivative for expression of *mrx1* (*ncgl0808*) | [[5](#_ENREF_25)] |
| pET15b | Expression vector with N-terminal hexahistidine affinity tag | Novagen |
| pET15b-*lpd* | pET15b derivative for expression of *lpd* (*ncgl0355*) | This study |

**References**

1. Tauch A, Kirchner O, Löffler B, Götker S, Pühler A, et al. Efficient electrotransformation of *Corynebacterium diphtheriae* with a mini-replicon derived from the *Corynebacterium glutamicum* plasmid pGA1. Curr Microbiol. 2002; 45: 362-367.
2. Si M, Long M, Chaudhry MT, Xu Y, Zhang P, et al. Functional characterization of *Corynebacterium glutamicum* mycothiol S-conjugate amidase. PloS One. 2014; 9:e115075.
3. Schafer A, Tauch A, Jager W, Kalinowski J, Thierbach G, et al. Small mobilizable multi-purpose cloning vectors derived from the *Escherichia coli* plasmids pK18 and pK19: selection of defined deletions in the chromosome of *Corynebacterium glutamicum*. Gene.1994; 145: 69-73.
4. Jakoby M, Ngouoto-Nkili CE, Burkovski A. Construction and application of new *Corynebacterium glutamicum* vectors. Biotechnol Tech.1999; 13: 437-441.
5. Si MR, Zhang L, Yang ZF, Xu YX, Liu YB, et al. NrdH redoxin enhances resistance to multiple oxidative stresses by acting as a peroxidase cofactor in *Corynebacterium glutamicum*. Appl Environ Microbiol. 2014; 80: 1750-62.
