# Supplementary material for: Ohr Protects Corynebacterium glutamicum against Organic Hydroperoxide Induced Oxidative Stress
Source: PLoS One. 2015 Jun 29;10(6):e0131634. doi: 10.1371/journal.pone.0131634 (PMC4486171; doi:10.1371/journal.pone.0131634)
Supplement: S2 Table — (DOCX) [file pone.0131634.s002.docx]

**Table S2. Primers used in this study.**

| **Primers** | **5’-3’ sequence** |  |
| --- | --- | --- |
| Ncgl0355-F | GGGAATTCCATATGGTGACTGAACATTATG (Nde I) | To generate  pET15b-*ncg1*0355 |
| Ncgl0355-R | CCGCTCGAGCTAGAAGTTGATCATGTGTCC (Xho I) |  |
| Ncgl0658-F | CCGGAATTCATGGCAAAGAGGATCGTAATTATC(EcoR I) | To generate  pET28a-*ncg1*0658 |
| Ncgl0658-R | CCCTCAGTTAGCCTAGATCATCATGTTG (Xho I) |  |
| Ncgl2126-F | CCGGAATTCATGGCGTTCTCCGTAGAGATGC(EcoR I) | To generate  pET28a-*ncg1*2126 |
| Ncgl2126-R | CGCGGATCCTTAGAGCTGCAGATCGCCTTC(BamHI) |  |
| Ohr-F | GGAAGATCTATGGCGATCGAGTCCATCGCGTAC (Bgl II) | To generat  pET28a-*ncg1*0023 |
| Ohr-R | ACGCGTCGACTTAAGCCTCTTCGTCGATGACTG (Sal I) |  |
| Ohr-C60S-R | GGTTACGCAGCC**TCT**TTCCACTCTGC | To generate  pET28a-*ohr::C60S* |
| Ohr-C60S-F | GGCAGAGTGGAA**AGA**GGCTGCGTAAC |  |
| Ohr-C124S-R | CGCACCAGGTG**TCC**CCGTATTCCAA | To generate  pET28a-*ohr::C124S* |
| Ohr-C124S-F | TTGGAATACGG**GGA**CACCTGGTGCG |  |
| Dohr-F1 | CGCGGATCCGGTCGGTCTGGTGAACTGGATTG (BamH I) | To generate pK18*mobsacB-ncgl0023* |
| Dohr-R1 | GCCGGTTGAGAGTGCTTCACTGG |  |
| Dohr-F2 | CCAGTGAAGCACTCTCAACCGGC |  |
| Dohr-R2 | ACGCGTCGACGGCTCTAGCAAACGCCGTACTTC (Sal I) |  |
| DsigH-F1 | TCCCCCGGGCGCGCATTTCTCGGGTTGGAG (SmaI) | To generate  pK18*mobsacB-ΔsigH* |
| DsigH- R1 | CCGAATTCGTGTCGAGCATCGTGGCAGTGCCTCCTCTTC |  |
| DsigH- F2 | CCGAATTCGTGTCGAGCATC |  |
| DsigH- R2 | ACGCGTCGACCACCGGTGCAGCGCAAGATG (SalI) |  |
| lacZY-F | GAAACACGTGAGTGGTCTACGCTCTAGAACTAGTATGA CCATGATTACGGATTC (SpeI) | To generate  *lacZY* fragment |
| lacZY-R | AAAACTGCAGTTAAGCGACTTCATTCACCTG (PstI) |  |
| P_ohr_-F | TCCCCCGGGCTCAGTGGACTCCTCGGCATTG (Sma I) | To generate pK18*mob*  *sacB*- P*_ohr_::lacZ* |
| P_ohr_-R | CTAGTCTAGACGCGATGGACTCGATCGCCATAGC (Xba I) |  |

Underlined sites indicate restriction enzyme cutting sites added for cloning. Letters in italic denote the mutation sites in overlap PCR for site-directed mutation.
